# Supplementary figures and images for: Integrated microbiology and metabolomics analysis reveal the fermentation process and the flavor development in cigar tobacco leaf
Source: Microbiol Spectr. 2025 Apr 24;13(6):e01029-24. doi: 10.1128/spectrum.01029-24 (PMC12131730; doi:10.1128/spectrum.01029-24)

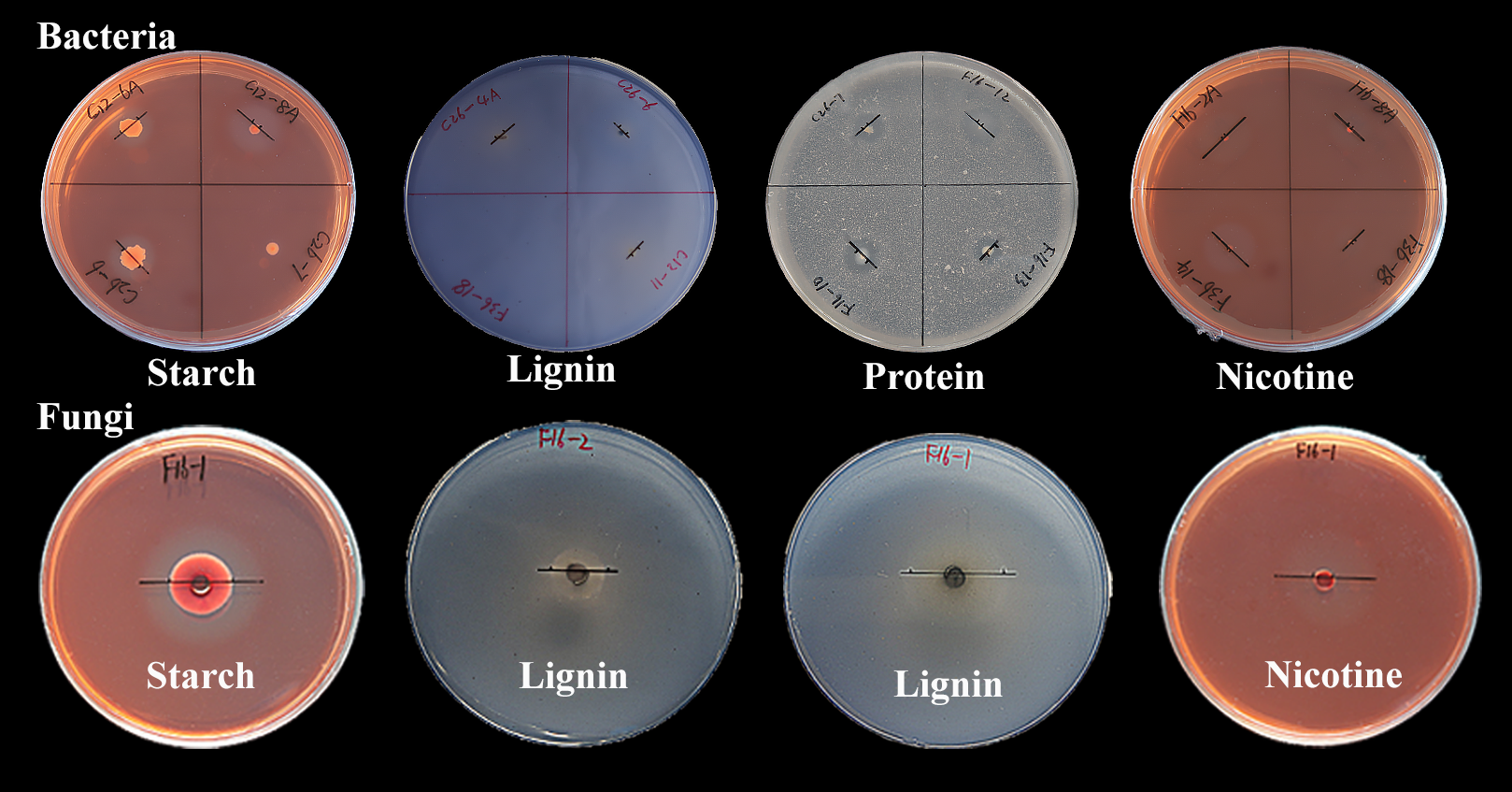

Supplement: Fig. S1 — Degradation circle size of culturable bacteria from cigar tobacco leaves. [file spectrum.01029-24-s0001.tif]

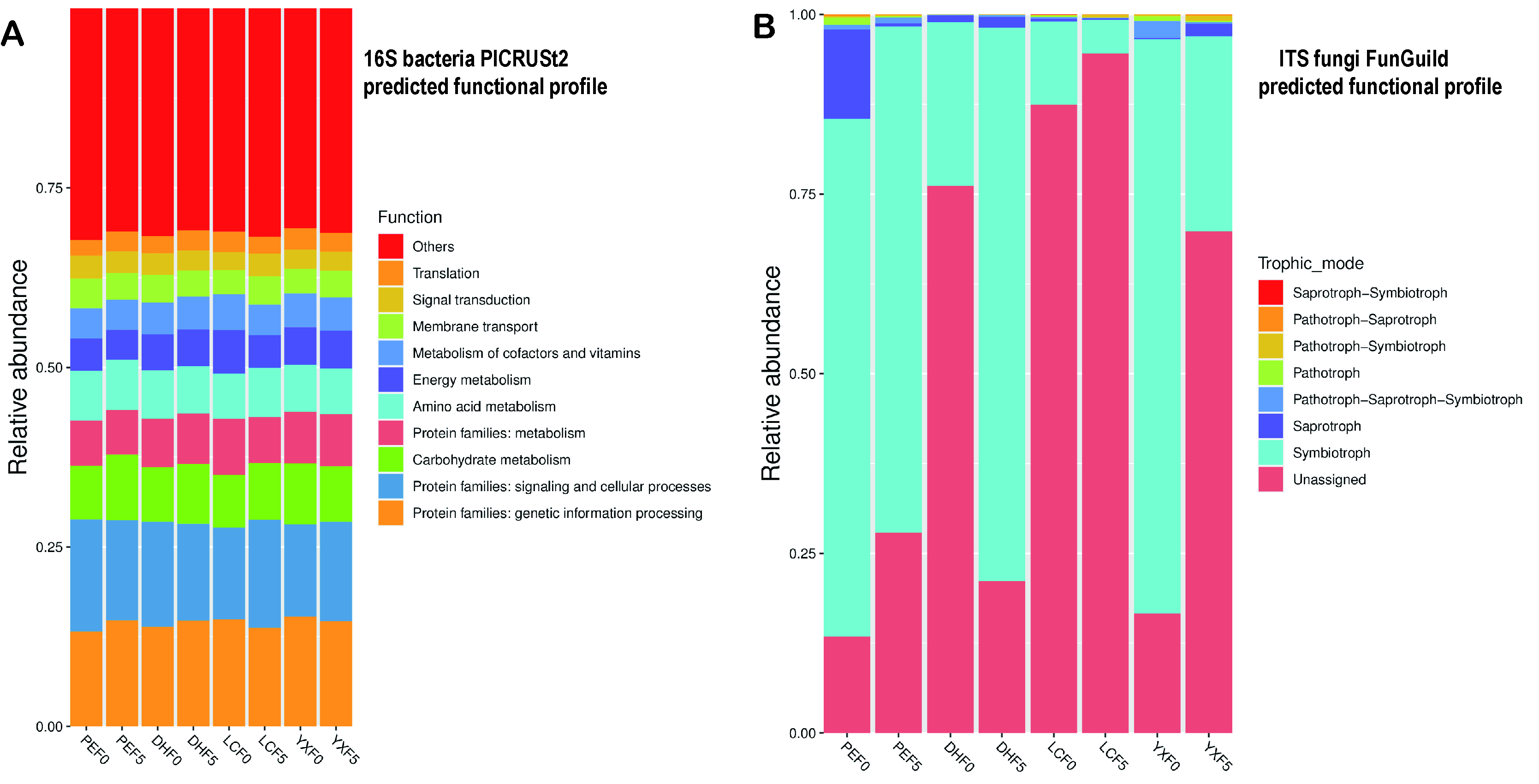

Supplement: Fig. S2 — Predicted functional profile of bacteria and fungi. [file spectrum.01029-24-s0002.tif]
