# Supplementary material for: Integrated microbiology and metabolomics analysis reveal the fermentation process and the flavor development in cigar tobacco leaf
Source: Microbiol Spectr. 2025 Apr 24;13(6):e01029-24. doi: 10.1128/spectrum.01029-24 (PMC12131730; doi:10.1128/spectrum.01029-24)
Supplement: Supplemental material — Legends for supplemental tables and figures. [file spectrum.01029-24-s0003.docx]

**Supplementary material legends**

Figure S1: Degradation circle size of culturable bacteria from cigar tobacco leaves

Figure S2: The predicted functional profile of bacteria and fungi.

Table S1: Quality control of high-throughput microbial amplicon sequencing.

Table S2: Classification of culturable bacteria and Fungi in cigar tobacco leaves.

Table S3: The significant differential metabolites summary of HS-SPME-GC-MS and UPLC-ESI-MS/MS.

Table S4: The spearman correlation of 16S bacteria genus and volatile metabolites.

Table S5: The spearman correlation of ITS fungi genus and volatile metabolites.

Table S6: The enriched KEGG metabolic pathway of non-volatile metabolites.
